# Characterization of the cHS4 insulator in mouse embryonic stem cells

**DOI:** 10.1002/2211-5463.12818

**Published:** 2020-03-19

**Authors:** Xi‐bin Lu, Yu‐han Guo, Wei Huang

**Affiliations:** ^1^ Core Research Facilities Southern University of Science and Technology Shenzhen China; ^2^ Forward Pharmaceuticals Limited Co. Shenzhen China; ^3^ Department of Biology Southern University of Science and Technology Shenzhen China

**Keywords:** cHS4, embryonic stem cell, insulator, *Nanog*, synthetic biological circuit, transposon

## Abstract

Synthetic biology circuits are often constructed with multiple gene expression units assembled in close proximity, and they can be used to perform complex functions in embryonic stem cells (ESCs). However, mutual interference between transcriptional units has not been well studied in mouse ESCs. To assess the efficiency of insulators at suppressing promoter interference in mouse ESCs, we used an evaluation scheme in which a tunable tetracycline response element promoter is connected to a constant *Nanog* promoter. The chicken hypersensitive site 4 (cHS4) insulator, widely used both for enhancer blocking and for barrier insulation *in vitro* and *in vivo*, was positioned between the two expression units for assessment. By inserting the cassette into various loci of the mouse ESC genome with *PiggyBac* transposon, we were able to quantitatively examine the protective effect of cHS4 by gradually increasing the transcriptional activity of the tetracycline response element promoter with doxycycline and then measuring the transcriptional activity of the *Nanog* promoter. Our results indicate that the cHS4 insulator has minimal insulating effects on promoter interference in mouse ESCs. Further studies show that the cHS4 insulation effect may be promoter specific and related to interaction with CCCTC‐binding factor‐mediated loop formation. In addition, we also compared DNA transposition and transgene expression with or without the cHS4 insulator using well‐established ESC reporters. The results indicate that cHS4 has no apparent effects on DNA transposition and transgene expression levels, but exerts modest protective effects on long‐term transgene silencing.

## Graphical abstract

Here we investigated the effect of the chicken hypersensitive site 4 (cHS4) insulator on *PiggyBac* transposon‐based vectors for gene transfer in mouse embryonic stem cells. The presence of the cHS4 insulator between two transcription units did not make any significant difference on suppression of promoter interference and transposition efficiency. Positioning of the cHS4 insulator at both ends of expressing units provided only a modest effect on preventing transgene silencing.

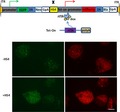

**Abbreviations**
CAG: cytomegalovirus enhancer and chicken beta‐actin promoter
cHS4: chicken hypersensitive site 4
CTCF: CCCTC‐binding factor
DMEM: Dulbecco’s modified Eagle’s medium
EGFP: enhanced GFP
ES: embryonic stem
ESC: embryonic stem cell
ITR: inverted terminal repeat
SLIC: sequence and ligation‐independent cloning
TRE: tetracycline response element

Stable transgene integration is often mediated by a virus, such as adenovirus or lentivirus. However, transposon‐based gene transfer is becoming popular due to its cargo capacity and simple manipulation procedure. Currently, *PiggyBac*, *Sleeping Beauty* and *Tol*2 are the three most commonly used transposon systems. Among the three systems, *PiggyBac* possesses several critical characteristics. First, it has high cargo capacity with which it is reported that up to 100 kb has been successfully transposed in the mouse genome [1]. Second, most reports showed that it has no transposase overproduction inhibition, a phenomenon possessed by *Sleeping Beauty* [2]. Last and most importantly, it can reexcise the inserted transposon without leaving a footprint by introducing transposase. Owing to its unique features, the *PiggyBac* system has been widely used in many applications, such as transgenesis in mice [3], gene transfer in embryonic stem cells (ESCs) [4, 5] and induced pluripotent stem cell  reprogramming [6].

Multiple gene expression cassettes with different gene regulatory elements were often assembled together to study the functions of synthetic gene circuits [7, 8]. In previous studies, it has been reported that transgene expressions were often mutually interfered when two transgenes driven by different promoters were coexpressed in a single plasmid, a phenomenon called *promoter suppression* or *transcriptional interference* [9, 10, 11]. In addition, transgene silencing or variability of expression was often observed with viral‐ or transposon vector‐mediated random integration because of the transgene copy number or integration sites [12, 13]. To avoid mutual interaction or transgene silencing, genomic insulators were often chosen to be inserted among transcriptional units or flanked at both ends of expressing cassettes. So far, several insulators or elements, such as the chicken hypersensitive site 4 (cHS4) insulator or scaffold or matrix attachment regions elements, have been found to play positive roles in enhancing transgene expression or blocking transgene silencing in previous studies [14, 15, 16]. The combination of HS4 and scaffold attachment region elements (chimeric insulator) was also reported to enhance transgene expression across different cell lines, such as human pluripotent stem cells, Chinese hamster ovary cells and 293T cells [17, 18, 19]. The most widely used is the cHS4 insulator, which possesses both barrier activity (blocking the spreading of heterochromatin from gene silencing) and enhancer blocking activity (segregating enhancer and promoter from interaction) [20, 21], and was often positioned among gene expression cassettes. Previous reports showed that cHS4 insulator could alleviate the interference of two promoters in lentiviral‐mediated transgenesis [22, 23]. However, the actual insulation effect in *PiggyBac*‐mediated transgenesis in mouse ESCs has not been characterized. In addition, when cHS4 insulator was inserted between an enhancer and gene expression cassette in the context of virus backbone, only modest enhancer‐blocking activity was observed [24].

To better understand the cHS4 insulator in protecting transcriptional interference in mouse ESCs, we constructed an evaluation cassette by putting an inducible tetracycline response element (TRE) promoter and an ESC marker gene *Nanog* promoter in tandem. We used mCherry and GFP to monitor their transcriptional activity, respectively. The cHS4 insulator was flanked either in the middle or both sides of the cassette. The constructs were cloned into a *PiggyBac* transposon vector to generate ES reporter cell lines. We quantitatively characterized cHS4 insulator in transcriptional blocking activity with the ESC lines. Although previous reports have shown that cHS4 insulator efficiently improves or stabilizes transgene expression in various cell types [25, 26, 27], negligible results were also reported. In the context of virus backbone, it even can reduce transduction efficiency and virus titers [24, 28]. Therefore, we also compared the transposition efficiency and transgene expression in the context of *PiggyBac* backbone with or without the cHS4 insulator in mouse ESCs.

## Materials and methods

### Plasmid construction

All of the plasmids are listed in Table 1. Specifically, PL451 plasmid was used as the original backbone. Two copies of core cHS4 (250 bp) was amplified from plasmid pEGFP‐N1‐Cha4 and designated as the HS4 insulator. 5′ and 3′ terminal repeat of *PiggyBac* transposon was amplified using PCR from the plasmid PB‐SB‐Neo (gift from P. Liu). The HS4 insulator was first inserted into the *Eco*RI and *Nhe*I sites of PL451 plasmid. Then the 5′ terminal repeat and HS4 insulator was cloned into the *Kpn*I and *Apa*I sites using three‐piece ligation. The 3′ terminal repeat and HS4 insulator was cloned into *Not*I and *BstX*I sites using the same method. The constructed plasmid was named pBX‐023. For construction of pBX‐066, pBX‐098 plasmid was cut with *Xho*I and *Mfe*I, pBX‐084 was cut with *Eco*RI and *Not*I separately, and then the purified 4.5‐ and 2.1‐kb inserts were cloned into *Xho*I‐ and *Not*I‐digested pBX‐023 using three‐piece ligation. For construction of pBX‐067, plasmid pBX‐098 was cut with *Xho*I and *Mfe*I, pBX‐084 was cut with *Nhe*I and *Not*I, and the purified 4.5‐ and 2.1‐kb inserts were cloned into *Xho*I and *Eco*RI‐, *Nhe*I‐ and *Not*I‐digested pBX‐023 sequentially. H19 insulator was amplified from pWhere plasmid (InvivoGen, San Diego, CA, USA) and cloned into *Nhe*I‐digested pBX67 plasmid using the sequence and ligation‐independent cloning (SLIC) method to construct pBX‐068 [29]. pBX‐088 was digested with *Age*I and *Not*I to construct pBX‐095, and then the purified 2.4‐kb insert was cloned into *Age*I‐ and *Not*I‐digested pBX‐059. The PB5 and PB3 fragment was amplified from pBX‐023, and the digested PCR products were cloned into *Kpn*I and *Spe*I‐, *Not*I‐ and *BstX*I‐digested pBX‐095 sequentially to construct pBX‐097. The plasmid *Nanog*‐EGFPnuc‐2A‐NeoloxP‐GpA was cut with *Xho*I and *Not*I, and cloned into *Xho*I‐ and *Not*I‐digested pBX‐023 to construct pBX‐099. pBX‐099 was digested with *Kpn*I and *Xho*I, *Not*I and *BstX*I to remove PB5‐HS4 and HS4‐PB3 cassette separately and replace with PB5 and PB3 amplified from PB‐SB‐Neo plasmid to construct pBX‐101. For construction of pBX‐115, pBX‐084 was digested with *Nhe*I and *Not*I, and the purified 2.1‐kb fragment was cloned into *Nhe*I‐ and *Not*I‐digested pBX‐101. Then pBX‐115 was digested with *Nhe*I and inserted with the HS4 insulator for construction of pBX‐116 using the SLIC method. The plasmid pBX‐103 was digested with *Nhe*I and *Xho*I to construct pBX‐118, the isolated 2.6‐kb DNA fragment was purified as insert, and then the insert was cloned into *Nhe*I‐ and *Xho*I‐digested pBX‐115. The HS4 insulator was amplified with PCR and digested with *Nhe*I to construct pBX‐117; then the purified PCR product was cloned into *Nhe*I‐digested pBX‐118 with the SLIC method.

**Table 1:** Plasmids summary.

Plasmid no.	Plasmid name	Insert/Enzyme/Fragment size	Parent plasmid/Enzyme/Fragment size
pBX‐023	PB5‐HS4‐MCS‐HS4‐PGk‐Neo‐GpA‐HS4‐PB3	NA	NA
pBX‐059	PB5‐HS4‐CAG‐tdTomatonuc‐2A‐BlaloxN‐GpA‐HS4‐PB3	NA	NA
pBX‐066	PB5‐HS4‐NanogEGFPnuc‐2A‐NeoloxP‐GpA‐TRE‐mCherrynuc‐2A‐BlaloxN‐GpA‐HS4‐PB3	pBX‐098, pBX‐084 / *Xho*I+*Mfe*I, *Eco*RI+*Not*I / 4.5K, 2.1K	pBX‐023 / *Xho*I+*Not*I / 4.7K
pBX‐067	PB5‐HS4‐NanogEGFPnuc‐2A‐NeoloxP‐GpA‐HS4‐TRE‐mCherrynuc‐2A‐BlaloxN‐GpA‐HS4‐PB3	pBX‐098, pBX‐084 / *Xho*I+*Mfe*I, *Nhe*I+*Not*I / 4.5K, 2.1K	pBX‐023 / *Xho*I+*Eco*RI, *Nhe*I+*Not*I / 7.1K, 9.8K
pBX‐068	PB5‐HS4‐NanogEGFPnuc‐2A‐NeoloxP‐GpA‐H19‐TRE‐mCherrynuc‐2A‐BlaloxN‐GpA‐HS4‐PB3	H19 / PCR from pWhere / 2K	pBX‐067 / *Nhe*I / 12K
pBX‐084	PB5‐HS4‐TRE‐mCherrynuc‐2A‐bla‐GpA‐HS4‐PB3	NA	NA
pBX‐088	PB5‐HS4‐TRE‐tdTomatonuc‐2A‐ ZeoloxM3‐GpA‐HS4‐PB3	NA	NA
pBX‐095	PB5‐HS4‐CAG‐tdTomatonuc‐2A‐ZeoloxM3‐GpA‐HS4‐PB3	pBX‐088 / *Age*I+*Not*I / 2.5K	pBX‐059 / *Age*I+*Not*I / 6.5K
pBX‐097	PB5‐CAG‐tdTnuc‐2A‐zeoloxM3‐GpA‐PB3	PB5, PB3 / PCR / 442 bp, 336 bp	pBX‐095 / *Kpn*I+*Spe*I, *Not*I+*BstX*I / 8K, 7.5K
pBX‐098	PB5‐HS4‐Nanog‐EGFPnuc‐2A‐NeoloxP‐GpA‐HS4‐PB3	NA	NA
pBX‐099	PB5‐HS4‐Nanog‐EGFPnuc‐2A‐Neo‐GpA‐HS4‐PB3	Nanog‐EGFPnuc‐2A‐NeoloxP‐GpA / *Xho*I+*Not*I / 7.7K	pBX‐023 / *Xho*I+*Not*I / 4.7K
pBX‐101	PB5‐Nanog‐EGFP‐2A‐Neo‐GpA‐PB3	PB5, PB3 / PCR / 442 bp, 336 bp	pBX‐099 / *Kpn*I+*Xho*I, *Not*I+*BstX*I / 8.7K, 8.3K
pBX‐103	PL452‐PGK‐mAmetrinenuc‐2A‐Neo‐GpA	NA	NA
pBX‐115	PB5‐Nanog‐EGFP‐2A‐Neo‐TRE‐mCherrynuc‐2A‐bla‐GpA‐PB3	pBX‐084 / *Nhe*I+*Not*I / 2.1K	pBX‐101 / *Nhe*I+*Not*I / 8.2K
pBX‐116	PB5‐Nanog‐EGFP‐2A‐Neo‐HS4‐TRE‐mCherrynuc‐2A‐bla‐GpA‐PB3	HS4 / PCR / 605 bp	pBX‐115 / *Nhe*I / 10K
pBX‐117	PB5‐PGK‐mAmetrinenuc‐2A‐Neo‐GpA‐HS4‐TRE‐mCherrynuc‐2A‐bla‐GpA‐PB3	HS4 / PCR / 605 bp	pBX‐118 / *Nhe*I / 9K
pBX‐118	PB5‐PGK‐mAmetrinenuc‐2A‐Neo‐GpA‐TRE‐mCherrynuc‐2A‐bla‐GpA‐PB3	pBX‐103 / *Nhe*I+*Xho*I / 2.6K	pBX‐115 / *Nhe*I+*Xho*I / 5.7K

### ESC culture and transfection

Mitomycin‐treated SNL DG5 cell [30] (established from our laboratory) was cultured in Dulbecco’s modified Eagle’s medium (DMEM) and 10% FBS, 0.1 mm GlutaMAX (Invitrogen, Carlsbad, CA, USA). The D3 ESC (gift from J. Yue) line was maintained in high‐glucose DMEM supplemented with 15% ES‐qualified FBS, 0.1 mm GlutaMAX, 1× nonessential amino acid, 0.1 mm 2‐mercaptoethanol, 1000 U·mL^−1^ LIF, 50 U·mL^−1^ penicillin and 50 μg·mL^−1^ streptomycin. To generate a stable reporter ESC line, we conducted Lipofectamine‐mediated transfection according to the constructions. When cell density reached about 60–70% confluence, the transfection was conducted at a ratio of 1 μg transposon DNA per 2.5 μL Lipofectamine 2000. The next day, the cells were split, and appropriate densities (normally, the stable transfection efficiency based on *PiggyBac* is 1%) were seeded into 6‐cm dishes. Forty‐eight hours after transfection, selection medium containing neomycin was added and selected for about 1 week; fresh medium was changed daily to remove the dead cells.

### Doxycycline treatment

To turn on the inducible TRE‐driven expression reporter, we added a final concentration of 10–1000 ng·mL^−1^ doxycycline into the medium. After 48–72 h of doxycycline treatment, samples were prepared for imaging and flow analysis. Nontreated samples were used as negative control.

### Flow cytometry

Cell samples to be analyzed were trypsinized with 0.25% TE for 3–5 min; then the reaction was stopped with DMEM containing 10% FBS. To reduce background influence from medium, we centrifuged and resuspended samples with 1× PBS. Samples were filtered and immediately analyzed with BD LSRFortessa Analyzer. Cell debris was excluded by gating with forward and side scatter. Normally, 2 × 10^4^–3 × 10^4^ events were collected for analysis with flowjo software (Emerald Biotech Co., Ltd, Hangzhou, China).

### Colony assay experiment

To compare transposition efficiency with or without the HS4 insulator, we transfected equal molar quantities of transposons with *PiggyBac* transposase using Lipofectamine 2000. After transfection, corresponding antibiotics were added into the medium the following day. After 6–8 days of drug selection, the cells were fixed with 4% paraformaldehyde and washed three times with 1× PBS. Then the fixed cells were stained with alkaline phosphatase substrate solution (System Biosciences, Palo Alto, CA, USA) for 10 min. The colony number was counted using imagej software (NIH, Bethesda, MD, USA).

### Data analysis

Statistical analysis for comparison of each set of experimental means was performed using graphpad prism 5.0 software (GraphPad Software Inc., San Diego, CA, USA). *t*‐Test was performed on the raw data obtained. A *P* value <0.05 was considered statistically significant.

## Results

### Generation of dual‐reporter ESC line based on the *PiggyBac* system

**Fig. 1:**
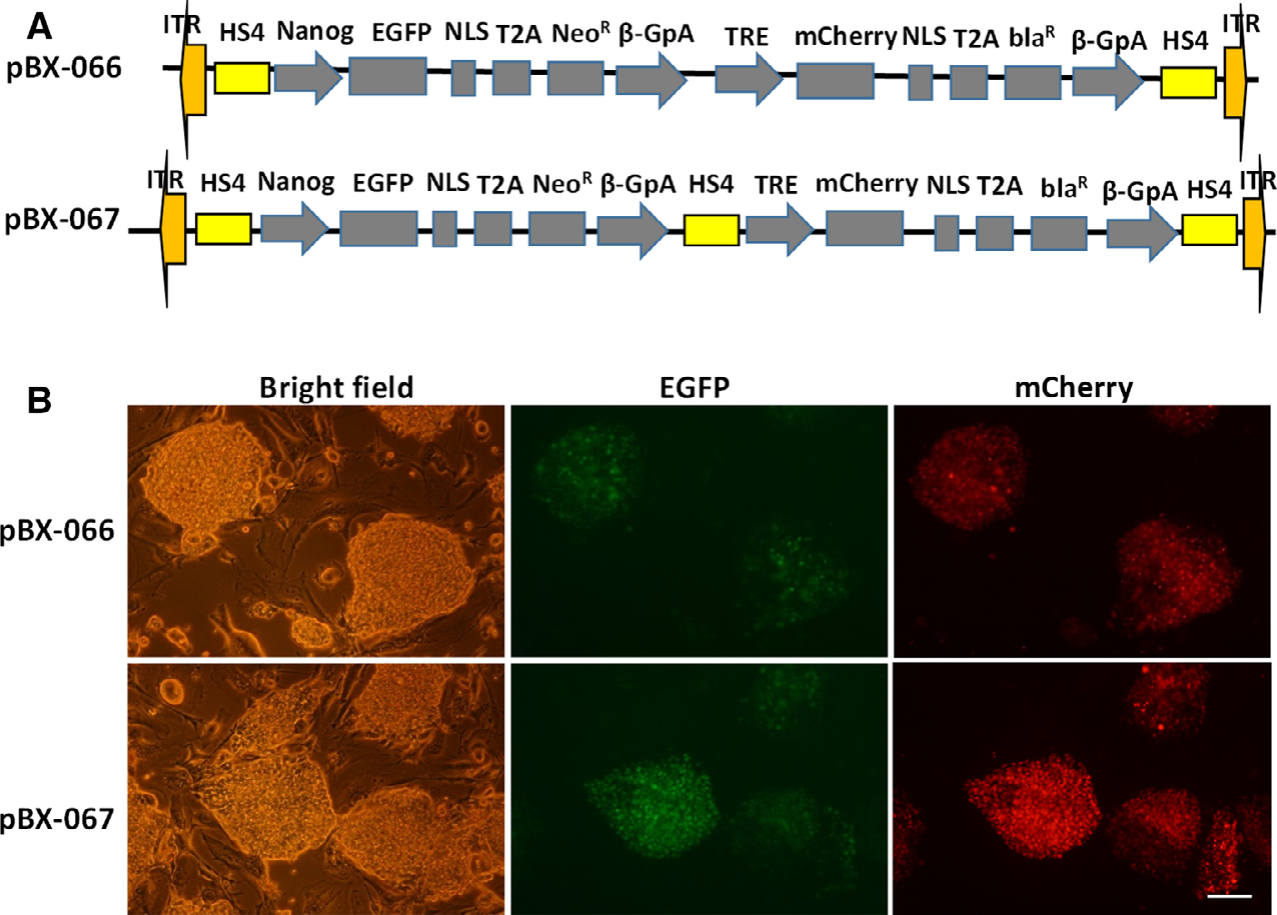
Generation of stable ESC line with two gene expression cassettes based on the *PiggyBac* system. (A) Schematic representation of pBX‐066 and pBX‐067 constructs. (B) Fluorescence imaging of generated stable ESC reporter with (insulated) or without (uninsulated) HS4 insulator. Images were taken with Nikon T2000 inverted microscope (Nikon corporation, Tokyo, Japan) under 10× objective; scale bar represents 200 µm. beta‐GpA, beta‐globin polyadenylation site; bla^R^, blasticidin resistance gene; Neo^R^, neomycin resistance gene; NLS, nuclear localization signal; T2A, 2A peptide from *Thosea asigna* virus.

The inverted terminal repeats (ITRs) of *PiggyBac* used in this study are 235 and 313 bp separately. To avoid position effects from the neighboring chromosomal environment, we also flanked two copies of the cHS4 insulator (designated as HS4) beside 5′ ITR and 3′ ITR separately. In this study, all fluorescence proteins were labeled with three copies of nuclear localization signal to facilitate imaging analysis if necessary. Compared with the internal ribosomal entry site, 2A peptide provides more consistent results for coexpression of two or more genes simultaneously [31, 32]. The shortest T2A peptide (54 amino acids) was adopted to mediate fluorescence protein and drug resistance gene coexpression. To avoid transcriptional interference, we positioned the HS4 insulator between two reporter expressing units. The final constructs were designated as pBX‐066 and pBX‐067 (Fig. 1A). To generate stable ESC clones, we cotransfected pBX‐066 (or pBX‐067) and helper plasmid *PiggyBac* transposase into the D3 ESC line. After transfection, G418 antibiotics were added into the medium and selected for 7 continuous days, and stable clones with GFP expression were circled under the inverted fluorescence microscope and then handpicked under bright field. Strong mCherry expression was also observed after doxycycline induction, which indicates that both cassettes can express efficiently (Fig. 1B).

### The HS4 insulator has limited protection upon promoter interference

**Fig. 2:**
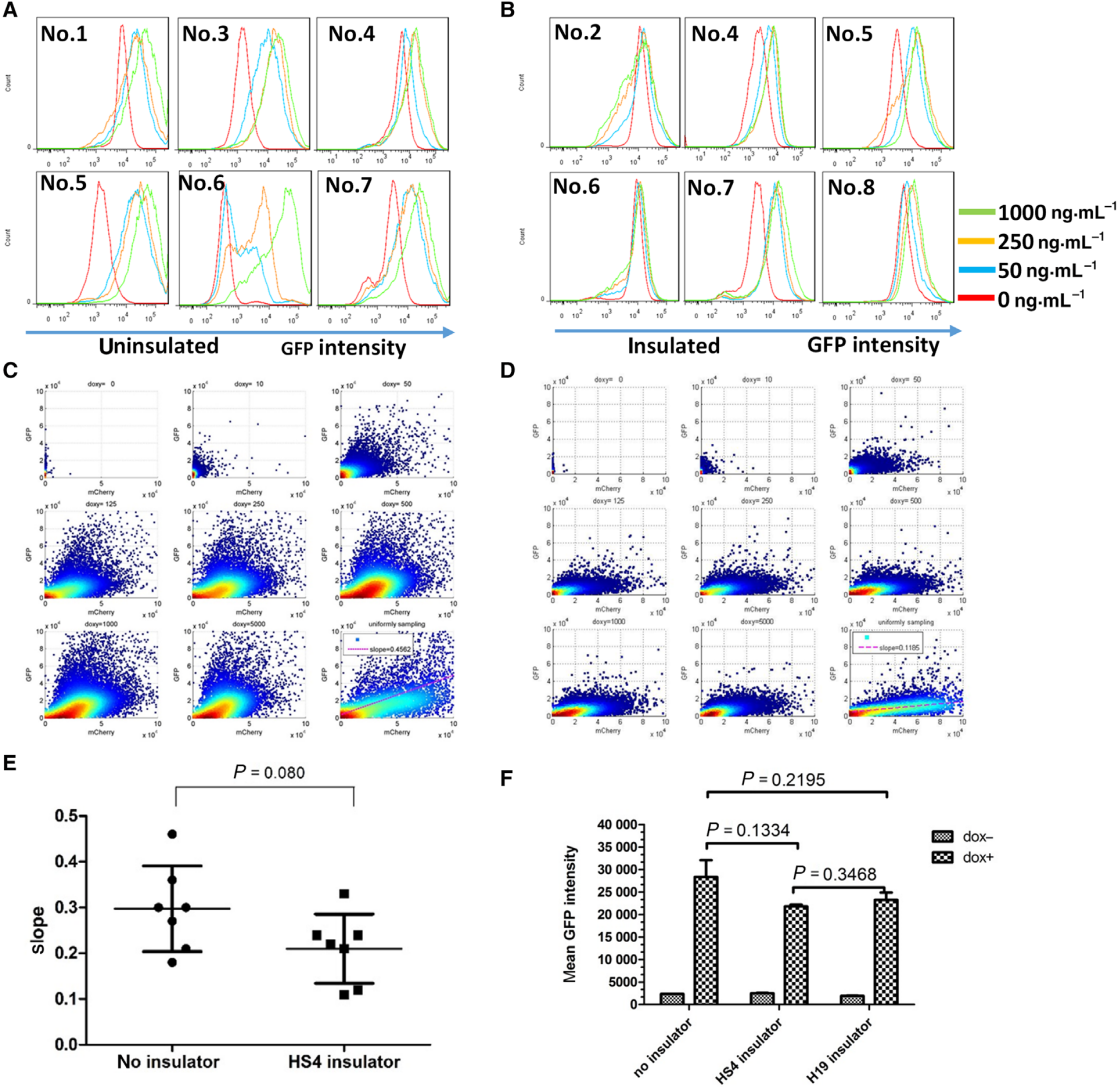
HS4 has minimal protection from neighboring promoter interference. (A, B) The histogram of GFP expression under 0, 50, 250 and 1000 ng·mL^−1^ doxycycline treatment. The generated stable ESC clones with (insulated) or without (uninsulated) HS4 insulator were treated with doxycycline under varied concentrations (50–1000 ng·mL^−1^) for 24 h; then GFP expression was analyzed with flow cytometry. The untreated samples were used as negative control. (C, D) The GFP and mCherry expressions in individual cells were measured based on flow cytometry, and the average slope was calculated using linear regression. (E) Statistical comparison of slope from the analyzed ESC clones (*n* = 7) in uninsulated and insulated groups. (F) Pool analysis of mean GFP intensity between HS4/H19 insulator and uninsulated group after doxycycline treatment. Data were analyzed by unpaired *t*‐test using graphpad software version 5.0. Bars represent means ± SEM (*n* = 2).

**Fig. 3:**
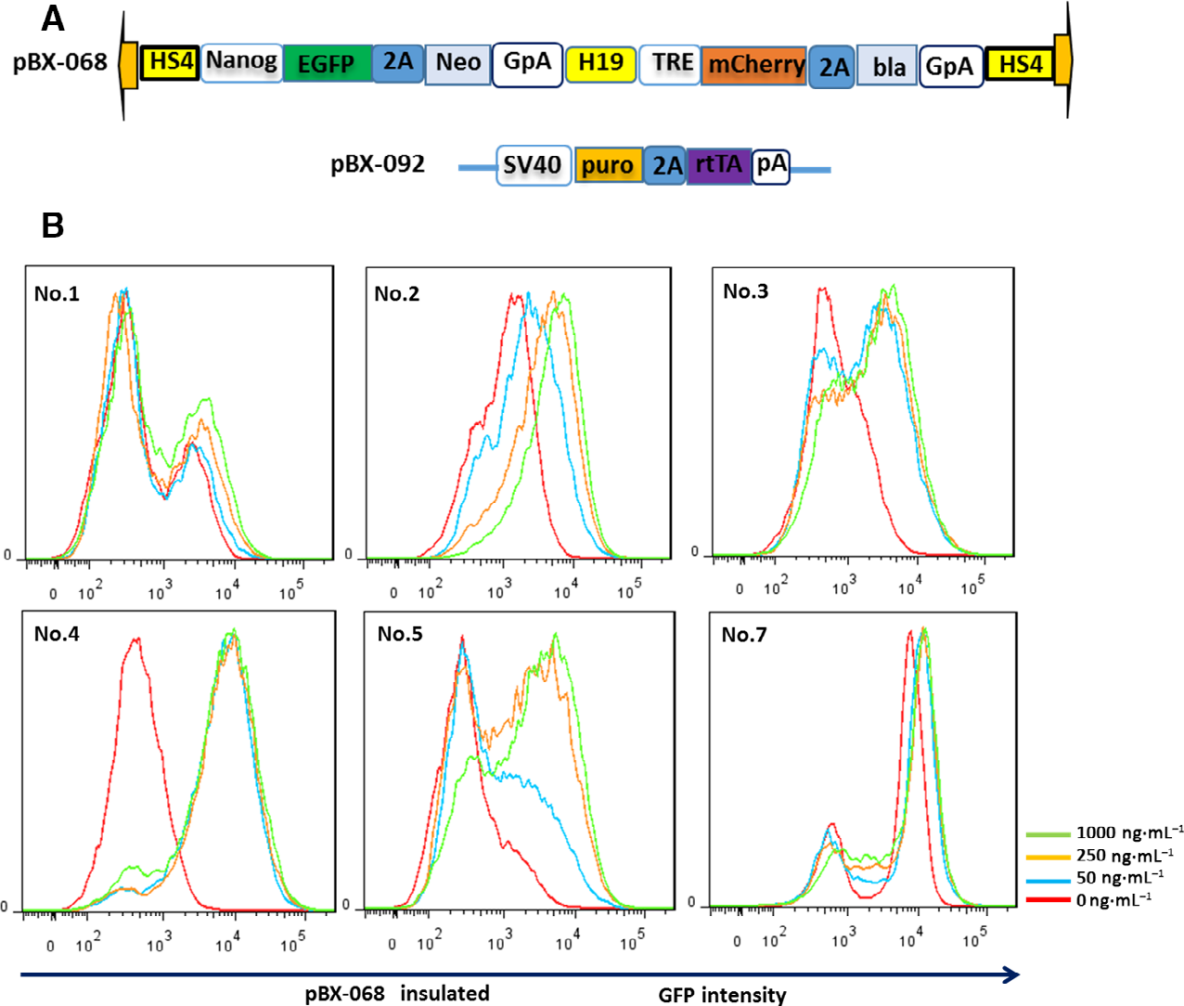
The H19 insulator performs a similar insulation effect on suppression‐inducible TRE promoter interference compared with the HS4 insulator. (A) Schematic representation of pBX‐068 construct. (B) The histogram of GFP expression under 0 (red), 50 (blue), 250 (orange) and 1000 ng·mL^−1^ (green) doxycycline treatment for pBX‐068. The red line is the histogram without doxycycline treatment as the negative control.

In many studies, the HS4 insulator was often integrated among different expression units to avoid mutual interferences [7, 8], but the actual insulation effect is not well studied in mouse ESCs. To measure the influence extent precisely and quantitatively, we integrated the HS4 insulator between two fluorescence reporter expressing units. The downstream mCherry fluorescence protein was driven by inducible TRE promoter, whereas the upstream GFP gene was driven by *Nanog* promoter. Thus, we can observe GFP expression level by tuning the downstream mCherry gene expression level with varying doxycycline concentrations (10–1000 ng·mL^−1^). By analyzing the upstream GFP expression based on flow cytometry, we found that *Nanog*‐driven GFP expression of all of the stable clones was increased to a different extent after doxycycline treatment in both uninsulated and insulated groups. The influence extent correlates with doxycycline concentration (Fig. 2A,B). To quantitatively compare the insulation effect, we measured with flow cytometry the GFP and mCherry expression under different doxycycline concentrations in individual cells. The slope, which quantitatively represents the influence of the TRE promoter transcriptional activity on *Nanog* promoter, was calculated. Among the analyzed clones, clone 4 in the insulated group and clone 6 in the uninsulated group were the two best representative examples (Fig. 2C,D). However, among the total of seven clones analyzed in each group, there are no statistically significant differences, although the insulated group performs better (Fig. 2E). This result indicated that the HS4 insulator has minimal protection against the inducible TRE promoter interference to the neighboring *Nanog*‐driven expression cassette. H19, another well‐characterized insulator that was found at the *insulin‐like growth factor 2* (*Igf2*)/*H19* locus, is also reported to have strong enhancer blocking activities [33]. To test the insulation effect of H19 insulator, we replaced HS4 with H19 insulator and established stable ESC lines following the same strategy (Fig. 3A). Compared with HS4, a similar phenomenon was observed after doxycycline treatment among the six stable clones analyzed (Fig. 3B). To reduce discrepancies caused by integration sites among different clones, we collected a pool of stable transfected cells (~104 clones) for each scenario and performed the same experiment and analysis with 1 μg·mL^−1^ doxycycline treatment. Although HS4 and H19 insulated groups performed better than the uninsulated group, the improvement is not statistically significant (Fig. 2F).

**Fig. 4:**
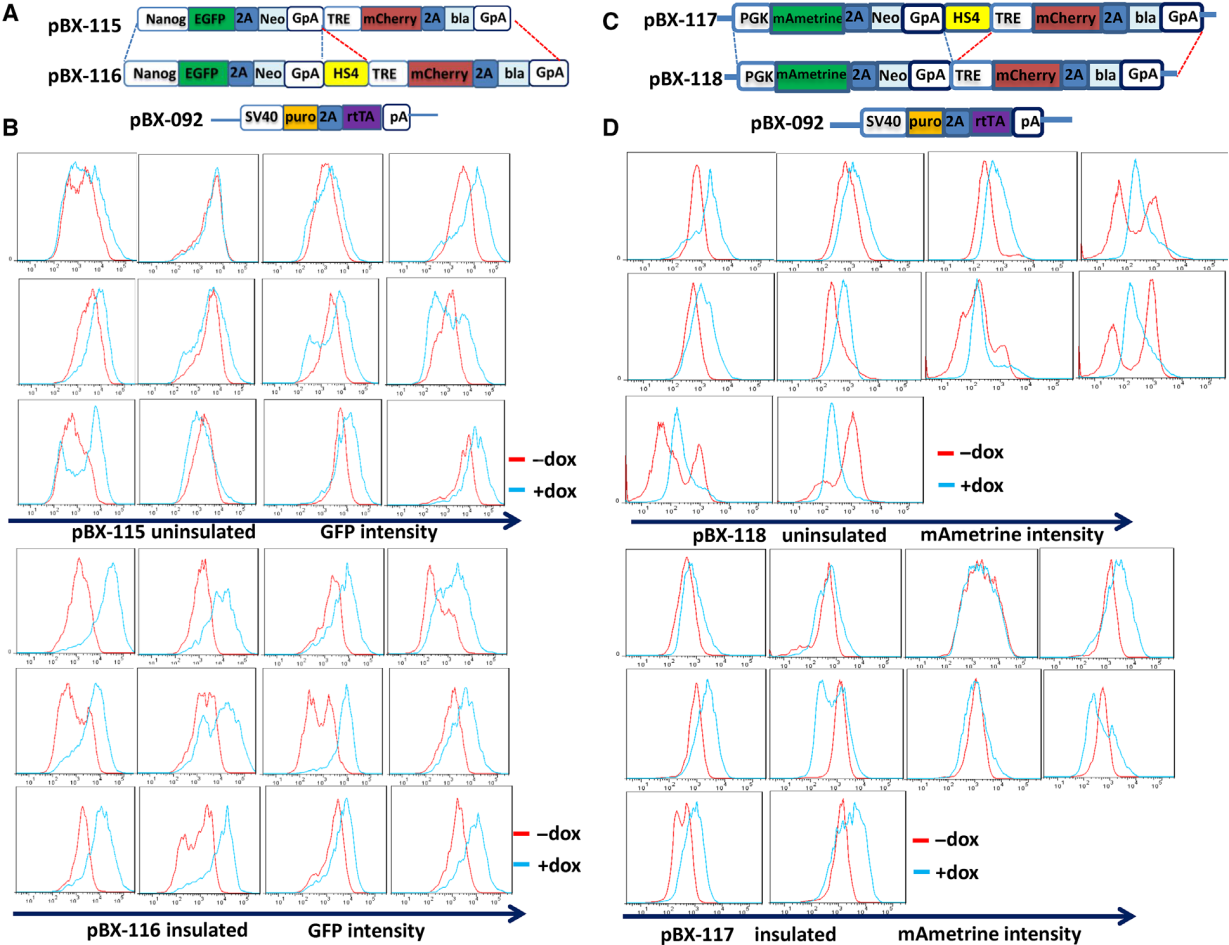
HS4 insulation effect on suppression from inducible promoter interference is promoter specific. (A) Schematic representation of pBX‐115 and pBX‐116 constructs. (B) The histogram of GFP expression under 1000 ng·mL^−1^ doxycycline treatment for pBX‐115 and pBX‐116. The red line represents the histogram without doxycycline treatment, and the blue line stands for the histogram after doxycycline induction. (C) Schematic representation of pBX‐117 and pBX‐118 constructs. (D) The histogram of GFP expression under 1000 ng·mL^−1^ doxycycline treatment for pBX‐117 and pBX‐118. The red line represents the histogram without doxycycline treatment, and the blue line represents the histogram after doxycycline induction.

Previous reports showed that the HS4 insulator could alleviate promoter interference by recruiting histone‐modifying complexes mediated by CCCTC‐binding factor (CTCF) and USF1 proteins [34], and recent reviews suggested that CTCF functional activity depends on the configuration or local geometry of DNA structure [20, 35]. In the initial design of constructs, HS4 was also flanked by two expression cassettes for avoiding transgene silencing. For example, three HS4 insulators that coexist in plasmid pBX‐067 may interact by CTCF‐mediated local geometry configuration. Thus, two HS4 insulators at both ends were removed to leave only the middle HS4 intact (Fig. 4A). Results showed that single HS4 still cannot protect the promoter interference effectively, whereas the control group with no HS4 insulator performed even better among the analyzed ESC clones analyzed (Fig. 4B). One study reported that CTCF is associated with several pluripotency marker genes, such as *Nanog* in human ESCs [36]. We suspected that the introduction of HS4 recruits CTCF protein binding, which may indirectly activate *Nanog* expression consequently. To test our hypothesis, we replaced the *Nanog* expression cassette with constant *phosphoglycerate kinase* (PGK) promoter‐driven gene expression unit (Fig. 4C). Using a similar method, we analyzed eight stable clones of uninsulated and insulated groups separately. The results show that PGK‐driven expressing cassette was modestly affected in both uninsulated and insulated groups compared with *Nanog*‐driven expressing unit (Fig. 4D). This result is consistent with our hypothesis but needs to be further characterized. Based on the results mentioned earlier, we conclude that the HS4 insulator has minimal protection effect on strong promoter interference. For some CTCF‐associated proteins, it may even have side effects for insulation protection.

### HS4 has no significant improvement on DNA transposition and transgene expression

**Fig. 5:**
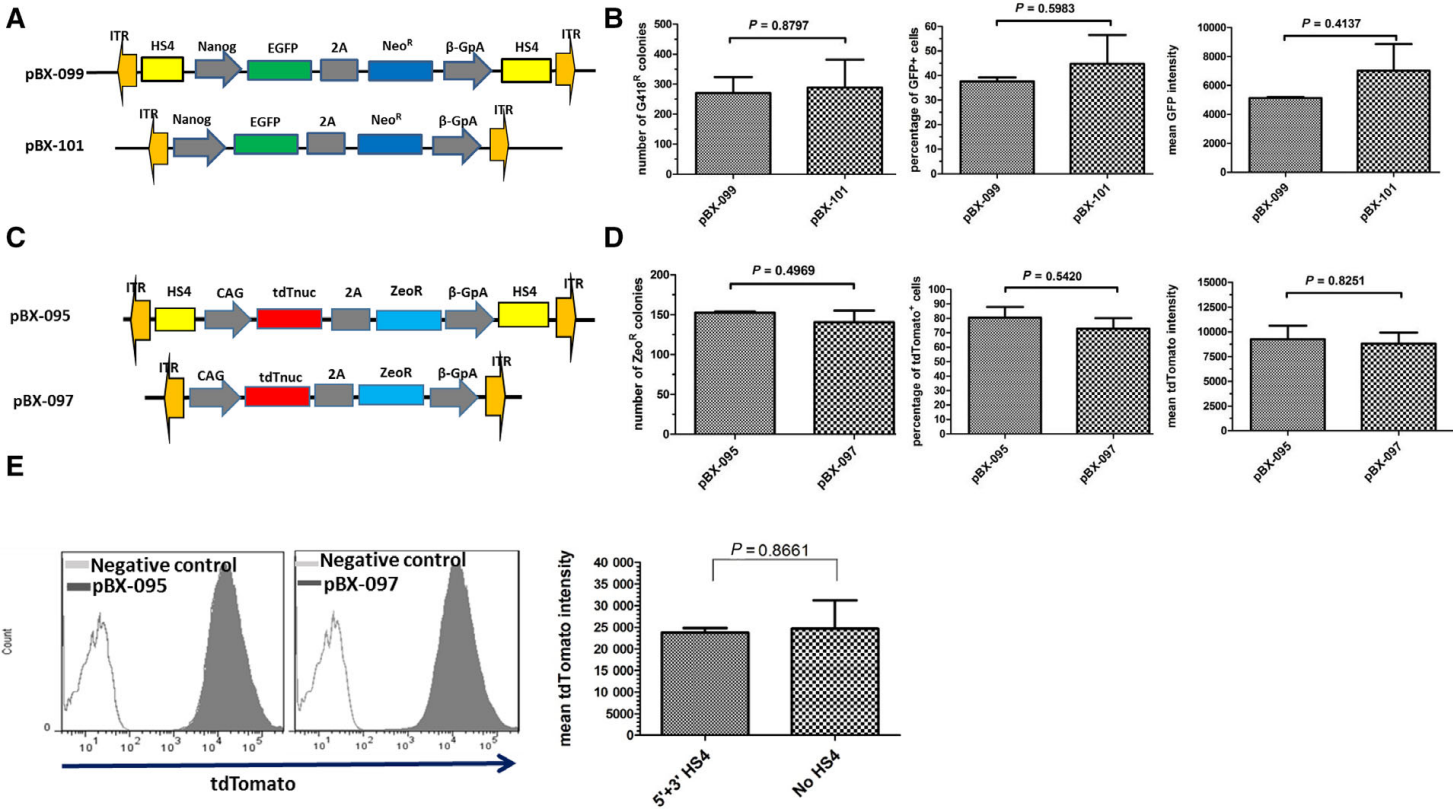
HS4 has no apparent improvement on DNA transposition and transgene expression. (A) Schematic representation of pBX‐099 and pBX‐101 constructs. (B) Comparison of DNA transposition and transgene expression between ESCs transfected with pBX‐099 and pBX‐101. Equal molar quantities of pBX‐099 and pBX‐101 were transfected into ESCs with the help of *PiggyBac* transposase. After about 1 week of G418 drug selection, cell colonies were either fixed for cell counting or trypsinized for flow analysis. Data were analyzed by unpaired *t*‐test using graphpad software version 5.0. Bars represent means ± SEM (*n* = 2). (C) Schematic representation of pBX‐095 and pBX‐097. (D) Comparison of DNA transposition and transgene expression between ESCs transfected with pBX‐095 and pBX‐097. Equal molar quantities of pBX‐095 and pBX‐097 were transfected into ESCs with the help of *PiggyBac* transposase. After about 1 week of zeocin selection, cell colonies were either fixed for cell counting or trypsinized for flow analysis. Data were analyzed by unpaired *t*‐test using graphpad software version 5.0. Bars represent means ± SEM (*n* = 2). (E) Comparison of transgene expression in 293T cells transfected with pBX‐095 and pBX‐097. Equal molar quantities of pBX‐095 and pBX‐097 were transfected into 293T cells with the help of *PiggyBac* transposase. After about 1 week of zeocin selection, cells were trypsinized for flow analysis. Data are shown as mean ± SEM (*n* = 2). tdTnuc, tdTomato fluorescence protein with nuclear localization signal; zeo^R^, zeocin resistance gene.

For commonly used gene delivery tools such as virus and transposon, the HS4 insulator was often placed next to the terminal repeats to stabilize or enhance transgene expression in different cell types [26, 37]. However, previous reports indicate that the inserted HS4 insulator could reduce virus titer or transduction efficiency in human hematopoietic cells [24]. To test whether the HS4 insulator could improve transposition efficiency and transgene expression in the context of *PiggyBac* transposon in mouse ESCs, we first established the *Nanog* reporter ESC line in which HS4 sequences were flanked in both sides of the reporter gene (Fig. 5A). After transfection, drug selection was maintained for 7 continuous days; then cells were fixed and stained for colony cell counting. Meanwhile, parallel cell populations were trypsinized for flow cytometry analysis. Cell colony assay indicates that transposition efficiency was similar in both groups. There is also no significant difference on the percentage of the GFP‐positive population and mean GFP expression intensity (Fig. 5B). This result demonstrated that the incorporation of HS4 does not enhance the transgene expression apparently, at least for short‐term effect in mouse ESCs. *Nanog* promoter is an ESC pluripotency marker that is sensitive to the culture environment. To exclude the possibility of culture influence, we generated a constant reporter in which tdTomato fluorescence protein is under the control of a constant cytomegalovirus enhancer and chicken beta‐actin promoter (CAG) (Fig. 5C). After the stable ESC line was established, cell colony number was calculated and protein expression was measured with flow cytometry analysis. Results indicated that there are also no significant differences between both groups (Fig. 5D). Previous reports showed that the HS4 insulator has a different or contradictory protection effect among different cell types [24, 26]. To test whether it is an ESC‐specific phenomenon, we transfected the CAG reporter constructs into the commonly used 293T cell line. The results also show there are no differences on transgene expression with or without HS4 insulator (Fig. 5E).

### HS4 has modest protection from long‐term transgene silencing

**Fig. 6:**
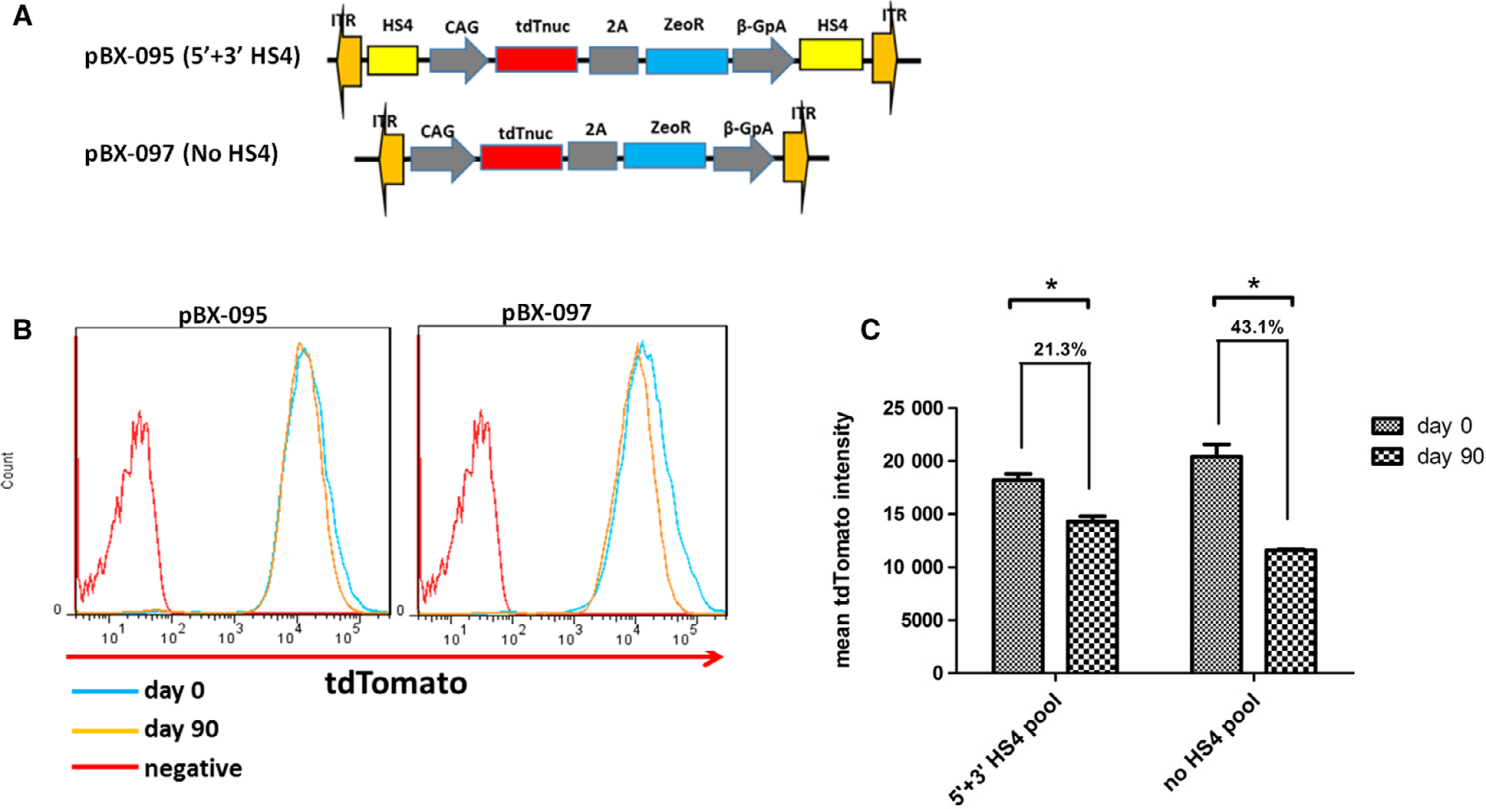
HS4 exhibits modest protection from long‐term transgene silencing. (A) Schematic representation of pBX‐095 and pBX‐097 constructs. (B) Comparison of transgene expression after 90 days of culture with (pBX‐095) or without (pBX‐097) the HS4 insulator. Generated ESC lines with (pBX‐095) or without (pBX‐097) the HS4 insulator were cultured for 90 continuous days; then the mean FP intensity was performed by flow cytometry. The red line on the left represents the histogram of untransfected ESCs. (C) The comparison of mean tdTomato fluorescence protein intensity for transfected pBX‐095 (5′ + 3′ HS4; *P* = 0.0378) and pBX‐097 (no HS4; *P* = 0.0182) at days 0 and 90. Data were analyzed by unpaired *t*‐test using graphpad software version 5.0. Bars represent means ± SEM (*n* = 2). *P < 0.05.

To observe the long‐term effect of HS4 insulator in protecting against transgene silencing, we used the plasmids pBX‐095 and pBX‐097 for generating stable ESC lines with constant fluorescence protein expression (Fig. 6A). The pools of CAG–tdTomato stable ESC lines with or without the HS4 insulator were generated and cultured for 90 days at the population level. Then the tdTomato transgene expression was measured with flow cytometry (Fig. 6B). The mean tdTomato protein expression levels at days 0 and 90 show that transgene expression are decreased in both groups, but the uninsulated reporter decreased significantly more compared with the insulated group (43.1% versus 21.3%), indicating that the HS4 insulator has an insulation effect in long‐term cell culture (Fig. 6C). Our result is consistent with a recent report, in which they use another constitutive EF1a promoter to drive transgene expression and test in a mouse prostate adenocarcinoma cell line, as well as human monocyte and erythroleukemia cell lines [38].

Taken together, our results indicate that the HS4 insulator has no apparent improvement in DNA transposition efficiencies and transgene expression levels in the context of *PiggyBac* backbone, both in mouse ESC and 293T cell lines, but it protects against transgene silencing in long‐term cell culture.

## Discussion

In this study, we analyzed the effect of the HS4 insulator on protecting from promoter interference in mouse ESCs based on a series of established ESC reporters. To quantitatively measure this activity, we introduced the HS4 insulator between a constant enhanced GFP (EGFP) reporter cassette and an inducible mCherry expression unit. Thus, we can readily quantify the transcriptional interference by measuring GFP expressions while tuning the downstream inducible gene expressions. This is a significant difference from the previous reports that mainly rely on constant promoters [39]. Under such circumstances, researchers had to generate several constructs with different promoters for comparison. Our results indicate that HS4 has limited insulation from the downstream inducible promoter. Similar results were also reported in human hematopoietic cells in the context of lentiviral vector [24]. This phenomenon is beyond expectation because the HS4 insulator has been integrated among two or more gene expression cassettes to avoid cross‐interaction in most studies [40]. Under certain circumstances, this may not affect the experimental results because the cassette flanking the insulator was usually an antibiotic resistance expression unit. However, if the flanking gene expression level is critical for determining cell fate, such as *Oct4* [41], this will pose unwanted outcomes. When we replaced with another commonly used H19 insulator, results showed that H19 performs similarly with HS4 on insulation effect. Previous studies indicated that CCCTC binding protein CTCF is involved in blocking promoter interaction [34]. It also binds to one of the footprints in a 250‐bp cHS4 sequence for enhancer blocking activity [42]. Another binding site of CTCF in vertebrate is the imprinted control region in the *Igf2*/*H19* locus, which thereby blocks the distal enhancer access to the IGF promoter [43, 44]. One of the most accepted mechanisms for CTCF‐mediated insulation is the three‐dimensional loop formation by CTCF and its binding sites. It was also proposed that the loop is formed by self‐interaction of CTCF molecules [45]. Previous studies in *Drosophila melanogaster* indicate that insulators could also interact with one another to organize chromatin loops [46, 47]. Furthermore, the interaction occurs in a pairwise manner that indicates that a pair of insulators will be neutralized and lose enhancer blocking activity, but not for single or odd insulators [48]. We do not know whether the HS4 insulator has a similar effect in mammalian cells. In our transposon vectors, we inserted two HS4 insulators flanking the double gene cassettes to avoid gene silencing. That means we have three HS4 insulators in the insulated reporter and two HS4 insulators in the uninsulated backbone. To exclude the possible influence of the HS4 insulator in both sides, we removed two HS4 insulators, leaving only one or none in the middle. Unexpectedly, results indicated that the uninsulated *Nanog*‐driven expressing unit performs even better than the insulated after the flanking HS4 insulators at both ends were removed. Several studies indicated that besides insulation function, CTCF‐mediated loop formation is also able to initiate transactivation with other transcription factors and RNA polymerase II [49, 50]. We speculate that when only one HS4 insulator leaves in the middle, the CTCF‐mediated protein complex may somehow activate the *Nanog* reporter in a specific way [51], because when we replaced the *Nanog* promoter with another constant PGK promoter, there are no significant differences with or without the HS4 insulator. Whether it is promoter specific or related to local DNA structure, it still needs to be further investigated.

Transcriptional silencing of transgene was often observed once integrated into the chromosome. Incorporation of chromatin insulators was one of the strategies to block the heterochromatin spreading. So far, the HS4 insulator has been successfully reported to improve the stability of transgene expression in different cell types, although the extent varies [24, 25, 52]. In the context of viral vectors, it is reported that the HS4 insulator could reduce the viral titers [24]. Few reports demonstrate the effect of the HS4 insulator on transposon‐based vectors, except a recent one focused on epithelium cell [53]. To study its effect on transposition and transgene expression in mouse ESCs, we used two ESC reporter lines: one is a pluripotency marker *Nanog*‐driven reporter and another is a constant promoter CAG‐driven reporter. The transposition assay based on the two reporters indicates there are no significant differences with or without the HS4 insulator. To some extent, the uninsulated *Nanog* reporter performs even better. Previous reports found that 5′ and 3′ terminal repeat contains promoter and enhancer activity separately, which may explain the observed phenomenon [54, 55]. To observe its protection effect from gene silencing, considering the instability of long‐term ESC culture, we had only long‐term culture based on our CAG reporter system. After 90 days of culture without antibiotics maintenance, both cassettes still showed high gene expression, which is consistent with a previous report [56], but transgene with HS4 insulator has less reduced gene expression. That indicates that HS4 could alleviate the long‐term silencing effect to some extent in *PiggyBac*‐based transgene strategy in mouse ESCs.

Overall, we had a quantitative characterization of the HS4 insulator in mouse ESCs. HS4 has limited protection against promoter interference. For a large cassette with more than one expression unit, careful design has to be taken into consideration. For *PiggyBac*‐based transgenesis in mouse ESCs, the HS4 insulator could be incorporated for mediating stable transgene expression in long‐term cell culture.

## Conflict of interest

The authors declare no conflict of interest.

## Author contributions

XL, YG and WH designed the experiments. XL performed the experiments and wrote the manuscript. XL, YG and WH analyzed the data. WH co‐revised the manuscript and helped in statistical analysis.
