# Correction to “Characterization of the cHS4 insulator in mouse embryonic stem cells”

**DOI:** 10.1002/2211-5463.70012

**Published:** 2025-05-05

## Abstract

Lu X, Guo Y, and Huang W, Characterization of the cHS4 insulator in mouse embryonic stem cells. *FEBS Open Bio.* 2020;10:644–656. https://doi.org/10.1002/2211-5463.12818

In the “Acknowledgements” section, the text “This work was supported by Shenzhen Science and Technology Innovation Commission Grants JCYJS20160530190702313 and Guangdong‐Hong Kong Joint Innovation Research Scheme (2016A050503010).” was incorrect. This should have read: “This work was supported by National Key R&D plan project (2019YFA0906002), Shenzhen Science and Technology Innovation Commission Grants JCYJS20160530190702313 and Guangdong‐Hong Kong Joint Innovation Research Scheme (2016A050503010).”

We apologize for this error.

Lu X, Guo Y, and Huang W, Characterization of the cHS4 insulator in mouse embryonic stem cells. *FEBS Open Bio.* 2020;10:644–656. https://doi.org/10.1002/2211-5463.12818

In the “Acknowledgements” section, the text “This work was supported by Shenzhen Science and Technology Innovation Commission Grants JCYJS20160530190702313 and Guangdong‐Hong Kong Joint Innovation Research Scheme (2016A050503010).” was incorrect. This should have read: “This work was supported by National Key R&D plan project (2019YFA0906002), Shenzhen Science and Technology Innovation Commission Grants JCYJS20160530190702313 and Guangdong‐Hong Kong Joint Innovation Research Scheme (2016A050503010).”

We apologize for this error.

